# Long Non-coding RNA Colon Cancer-Associated Transcript-1 Promotes Migration, Invasion, and Epithelial Mesenchymal Transition of Lung Adenocarcinoma by Suppressing miR-219-1

**DOI:** 10.3389/fgene.2020.00929

**Published:** 2020-10-16

**Authors:** Wenbo Wang, Zhiliang Hou, Chengcai Wen, Liyue Ge, Lili Ge

**Affiliations:** ^1^Department of Thoracic Surgery, Henan Provincial Chest Hospital, Zhengzhou, China; ^2^Huai’an Second People’s Hospital and The Affiliated Huai’an Hospital of Xuzhou Medical University, Huai’an, China; ^3^Department of Oncology, Huai’an Second People’s Hospital and The Affiliated Huai’an Hospital of Xuzhou Medical University, Huai’an, China; ^4^Department of Clinical Laboratory, Huai'an Second People’s Hospital and The Affiliated Huai’an Hospital of Xuzhou Medical University, Huai’an, China

**Keywords:** lung adenocarcinoma, colon cancer-associated transcript-1, miR-219-1, epithelial-mesenchymal transition, invasion

## Abstract

Previous evidence suggests that long non-coding colon cancer-associated transcript-1(CCAT1) plays a pivotal role in the progression of a variety of tumors. However, little is known about its role in lung adenocarcinoma (LAD). In this study, we found LAD tissue samples had a higher expression of CCAT1 but a lower expression of miR-219-1 compared to their adjacent non-tumor tissues. CCAT1 negatively regulated the expression of miR-219-1. miR-219-1 suppressed the proliferation of A549 and H1299 cells. Knockdown of CCAT1 inhibited the proliferation, migration, and invasion of A549 and H1299 cells, which were reversed by the miR-219-1 inhibitor. CCAT1 knockdown increased the expression of E-cadherin but decreased the expressions of N-cadherin and vimentin, which were restored by the miR-219-1 inhibitor. *In vivo*, knockdown of CCAT1 suppressed the tumor growth of LAD xenografts, which were rescued by the inhibition of miR-219-1. In summary, our findings suggested that CCAT1 promotes the progression of LAD *via* sponging miR-219-1, providing a potential therapeutic target for LAD.

## Introduction

Lung cancer is the leading cause of cancer-related death in China and worldwide (Bray et al., 2018). Of note, lung adenocarcinoma (LAD) is the most common subtype of non-small-cell lung cancer with high rates of mortality and metastasis (Travis et al., 2015). Despite the advances in therapeutic strategies in the last few decades, the prognosis of LAD patients is still unsatisfying (Miller et al., 2016). LAD can spread to lymph nodes, contralateral lung, and distant organs because of early invasion and metastasis (Guan et al., 2019). Efforts in uncovering the molecular mechanisms of LAD’s aggressiveness would facilitate the development of novel and effective therapeutic targets or prognostic biomarkers.

Long non-coding RNAs (lncRNAs) are a class of non-protein-coding transcripts longer than 200 nucleotides in length. Dysregulation of some lncRNAs promotes cellular processes related to cancer, including proliferation, invasion, metastasis, apoptosis, and drug resistance (Lu et al., 2017; Guan et al., 2019; Hu et al., 2019; Wang et al., 2019). Therefore, lncRNAs are considered a group of promising markers for predicting cancer prognosis and developing new therapeutic strategies. LncRNA colon cancer-associated transcript-1(CCAT1) was significantly upregulated in colon cancer tissue and cells (Nissan et al., 2012). Recently, CCAT1 has been studied in various cancers (Guo and Hua, 2017; Shi et al., 2017; Han et al., 2019; Hu et al., 2019; Li G. et al., 2019). CCAT1 plays a role in promoting the proliferation and invasion of cervical cancer by regulating the miR-181a-5p/MMP14 axis (Shen et al., 2019) and promotes endometrial carcinoma progression by miR-181a-5p (Yu et al., 2019). CCAT1 was also found to be an oncogene in nasopharyngeal carcinoma (Dong et al., 2018).

Silencing CCAT1 suppressed the proliferation, migration, and invasion and epithelial-mesenchymal transition (EMT) in NSCLC (Dong et al., 2018). However, the roles of CCAT1 in the pathogenesis of LAD remained mostly unknown. In this study, we observed significant upregulation of CCAT1 in LAD tissues compared to adjacent non-tumor tissues. The expression of CCAT1 was negatively associated with the expression of miR-219-1. CCAT1 promoted the growth of LAD xenografts *via* regulating miR-219-1. This study unveiled a potential therapeutic target for treating LAD.

## Materials and Methods

### Patient Samples

Twenty pairs of tumor and adjacent non-tumor tissues were collected from patients who received surgery for LAD at the Henan Provincial Chest Hospital between June 2016 and June 2018. Immediately after surgery, these tissues were snap-frozen using liquid nitrogen and were subsequently stored at a temperature of −80°C for future RNA extraction. No patients received radiotherapy or chemotherapy before surgery in this study. We obtained informed consent from all patients. The study was approved by the Ethics Committee of the Henan Provincial Chest Hospital.

### Cell Culture and Transfection

Two human LAD cell lines (A549 and H1299) were cultured in RPMI1640 medium supplemented with 10% fetal bovine serum (10099141, Invitrogen), 100 U/ml penicillin, and 100 μg/ml streptomycin (Invitrogen). A549 and H1299 are widely used as models of LAD. The condition was set as 5% CO_2_ at 37°C in a humidified incubator. The short hairpin RNAs against CCAT1 (CCAT1 shRNA and CCAT1 shRNA-2), control (shRNA NC), miR-219-1 mimic, and miR-219-1 inhibitor were designed by the Genepharma Co., Ltd. (Shanghai, China). Cell transfection was carried out using Lipofectamine 3000 (Invitrogen, CA, United States) in line with the manufacturer’s instructions. Quantitative real-time PCR (qRT-PCR) was used to confirm the efficiency of transfection. The cells were harvested for analysis 48 h after transfection.

### Luciferase Reporter Assays

The mutant (miR-219-1-MUT) and wild (miR-219-1-WT) types of miR-219-1 were cloned into the plasmid pGL3 (Promega). The CCAT1 luciferase vector was co-transfected with miR-219-1, miR-219-1-MUT, or miR-219-1-WT into HEK-239 T cells. After 48 h of transfection, the luciferase reporter assay was analyzed using the GloMax Multi Plus (Promega).

### RNA Extraction and Quantitative Real-Time PCR

Total RNA was extracted from the LAD tissues or cultured cell lines using the TRIzol reagent (15596018, Invitrogen, Thermo Fisher Scientific, United States). Total RNA was reverse transcribed to cDNA using the M-MLV reverse transcriptase (M1705, Promega, Madison, WI, United States). Real-time PCR analyses were performed with the SYBR Green system (Q221, Vazyme Biotech Co, Nanjing, China). The relative expression was calculated using the comparative threshold cycle method. Results were normalized to the expression of GAPDH for CCAT1 or U6 for miR-219-1, E-cadherin, N-cadherin, and vimentin.

### Cell Proliferation

We used the cell counting kit-8 (CCK-8; CK04, Dojindo, Kumamoto, Japan) to assess cell viability. Briefly, LAD cells transfected with shRNA NC, CCAT1 shRNA, or the miR-219-1 inhibitor were seeded in 96-well plates (1 × 10^4^ cells/well). Incubation lasted 1 h at 37°C in 5% CO_2_. Changes in cell viability were measured at 0, 1, 2, 3, 4, and 5 days. The absorbance at 450 nm was performed with a microplate reader Model 680 (Bio-Rad, CA, United States). All experiments were performed in triplicate.

### Wound-Healing Assay

Following transfection, we seeded cells in six-well plates (4 × 10^5^ cells/well) and cultured them until they reached approximately 90% confluence. Cell monolayers were scratched using a 200 μl pipette tip and were then cultured for 48 h. The scratched areas were photographed with the help of an inverted microscope and digital imaging system (Olympus, Japan) at 0 and 24 h.

### Cell Invasion Assay

The capacity of cell invasion was determined using 24-well Transwell chambers (3374, Corning, United States). Forty-eight hours post-transfection, we seeded the cells at a density of 5 × 10^4^ into the upper chamber of an insert coated with Matrigel (BD biosciences, United States) and filled the wells with 500 μl RPMI1640 containing 10% FBS. After incubation at 37°C for 48 h, we removed the cells on the upper side of the membrane with clean cotton swabs, fixed the cells on the underside with 1% formaldehyde solution for 15 min, and stained them with 0.1% of crystal violet for 15 min. Ten fields randomly selected were viewed under an inverted microscope. Experiments were conducted in triplicate.

### Xenograft Model

Male nude mice (8 weeks old) were purchased from the Model Animal Research Center of Nanjing University. The mice were housed under standard conditions with free access to food and water. The nude mice were randomly divided into six groups (six per group): A549 + shRNA NC, A549 + CCAT1 shRNA, A549 + CCAT1 shRNA + miR-219-1 inhibitor, H1299 + shRNA NC, H1299 + CCAT1 shRNA, and H1299 + CCAT1 shRNA + miR-219-1 inhibitor. An amount of 1 × 10^7^ cells were subcutaneously injected into the right rear limb of each mouse. The mice were weighed and the xenografts were measured every 3 days. The tumor volume was calculated as follows: tumor volume = (length × width^2^)/2. After 13 days, the mice were sacrificed, and the xenografts were harvested for further experiments. All procedures were approved by the Animal Care and Use Committee of the Huai’an Second People’s Hospital.

### Western Blot Analysis

The proteins of LAD tissues and cell lines were extracted with RIPA lysis buffer (KGP702, KeyGene Biotech, Nanjing, China). The protein concentration was measured using the Pierce™ BCA Protein Assay Kit (23227, Thermo Scientific™, United States). We separated equal amounts of protein (60ug) by 10% SDS-PAGE and transferred them to PVDF membranes. Firstly, we blocked the membranes using 5% non-fat milk in TBS Tween 20 (TBST). Then we incubated the membranes with primary antibodies in 5% bovine at 4°C overnight. The primary antibodies included E-cadherin (4A2C7, Invitrogen), N-cadherin (3B9, Invitrogen), and vimentin (V9, Invitrogen). The GAPDH antibody was used as a control. The membranes were washed three times with TBST and were incubated with horseradish peroxidase-conjugated secondary antibodies. The bands were measured using the Enhanced Chemiluminescence Kit (GE Healthcare, Chicago, IL, United States).

### Statistical Analysis

Statistical analysis was performed using SPSS version 22.0 (SPSS, IBM, United States). Differences between groups were compared by two-tailed Student’s *t*-test. Correlation between CCAT1 and miR-219-1 was calculated with Spearman correlation analysis. Data are shown as means and standard deviations (SD). Differences were considered statistically significant if *p* < 0.05. ^*^p < 0.05; ^**^p < 0.01.

## Results

### CCAT1 Is Upregulated in LAD Tissues

**Figure 1:**
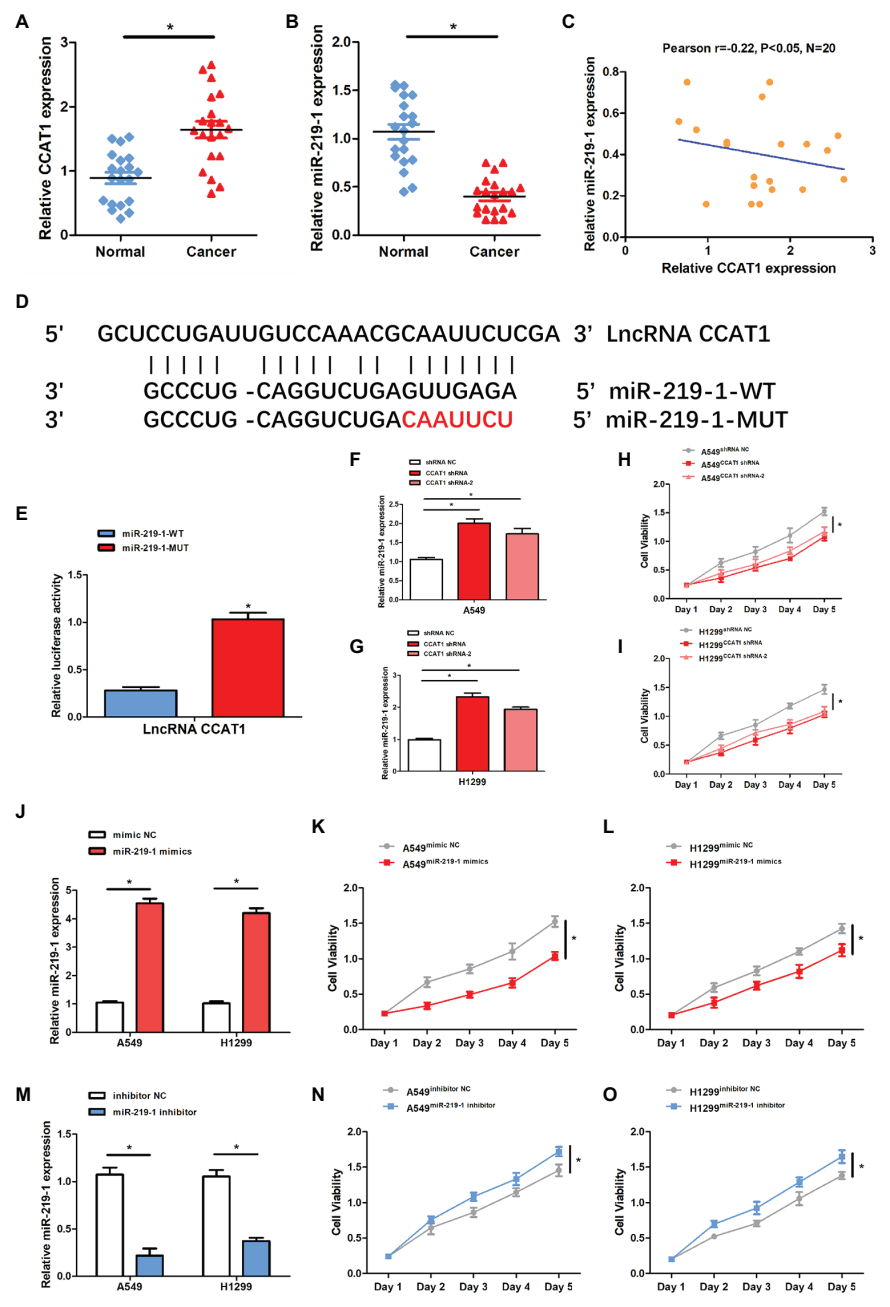
The expression of colon cancer-associated transcript-1(CCAT1) was negatively related to the miR-219-1 expression. **(A)** Relative CCAT1 expression in human lung adenocarcinoma (LAD) and adjacent non-tumor tissues. **(B)** Relative miR-219-1 expression in human LAD and adjacent normal tissues. **(C)** An inverse correlation between CCAT1 and miR-219-1 expressions in LAD tissue samples. **(D)** Bioinformatics analysis was performed to identify miR-219-1 binding sites in CCAT1. **(E)** A luciferase reporter assay was used to examine the relationship between CCAT1 and miR-219-1. **(F,G)** Relative miR-219-1 expression in A549 and H1299 cells transfected with CCAT1 shRNA and CCAT1 shRNA-2. **(H,I)** Cell viability of A549 and H1299 transfected with CCAT1 shRNA and CCAT1 shRNA-2. **(J)** Relative miR-219-1 expression in A549 and H1299 transfected with the miR-219-1 mimic and NC. **(K,L)** Cell viability of A549 and H1299 transfected with the miR-219-1 mimic and NC. **(M)** Relative miR-219-1 expression in A549 and H1299 transfected with the miR-219-1 inhibitor and NC. **(N,O)** Cell viability of A549 and H1299 transfected with the miR-219-1 inhibitor and NC. Data are shown as means ± SD. ^*^p < 0.05.

qRT-PCR was conducted to examine the relative expression of CCAT1 and miR-219-1 in 20 pairs of cancer and corresponding adjacent non-tumor tissues from patients with LAD. We found that lncRNA CCAT1 was significantly upregulated in LAD tissues compared to adjacent non-tumor tissues (Figure 1A). At the same time, the expression of miR-219-1 was significantly lower in LAD tissues compared to adjacent non-tumor tissues (Figure 1B). We also found that CCAT1 was negatively correlated with miR-219-1 (Figure 1C, Pearson’s *r* = −0.22, *p* < 0.05). To determine the relationship between CCAT1 and miR-219-1, bioinformatics analysis identified a potential miR-219-1 binding site in CCAT1 (Figure 1D).

Next, we used the luciferase reporter assay to validate the binding capability of miR-219-1 to CCAT1. We observed that the luciferase activity was reduced in cells transfected with miR-219-1-WT (Figure 1E). After transfection with CCAT1 shRNA and CCAT1 shRNA-2, the expression of miR-219-1 was significantly increased in both A549 and H1299 cells (Figures 1F,G). Moreover, knockdown of CCAT1 markedly suppressed the proliferation of A549 and H1299 cells (Figures 1H,I). These results suggested that CCAT1 promoted the proliferation of A549 and H1299 cells and negatively regulated miR-219-1.

Then we examined the effects of miR-219-1 in A549 and H1299 cells using the miR-219-1 mimic and inhibitor. We confirmed that the expressions of miR-219-1 in A549 and H1299 were successfully upregulated by the miR-219-1 mimic (Figure 1J). The cell viability of A549 and H1299 was significantly suppressed by the overexpression of miR-219-1 (Figures 1K,L). Additionally, the expressions of miR-219-1 in A549 and H1299 were inhibited by the miR-219-1 inhibitor (Figure 1M). The inhibition of miR-219-1 significantly increased the cell viability of A549 and H1299 (Figures 1N,O). These results suggested that miR-219-1 suppressed the proliferation of A549 and H1299 cells.

### Knockdown of CCAT1 Inhibits LAD Cell Proliferation *via* miR-219-1

**Figure 2:**
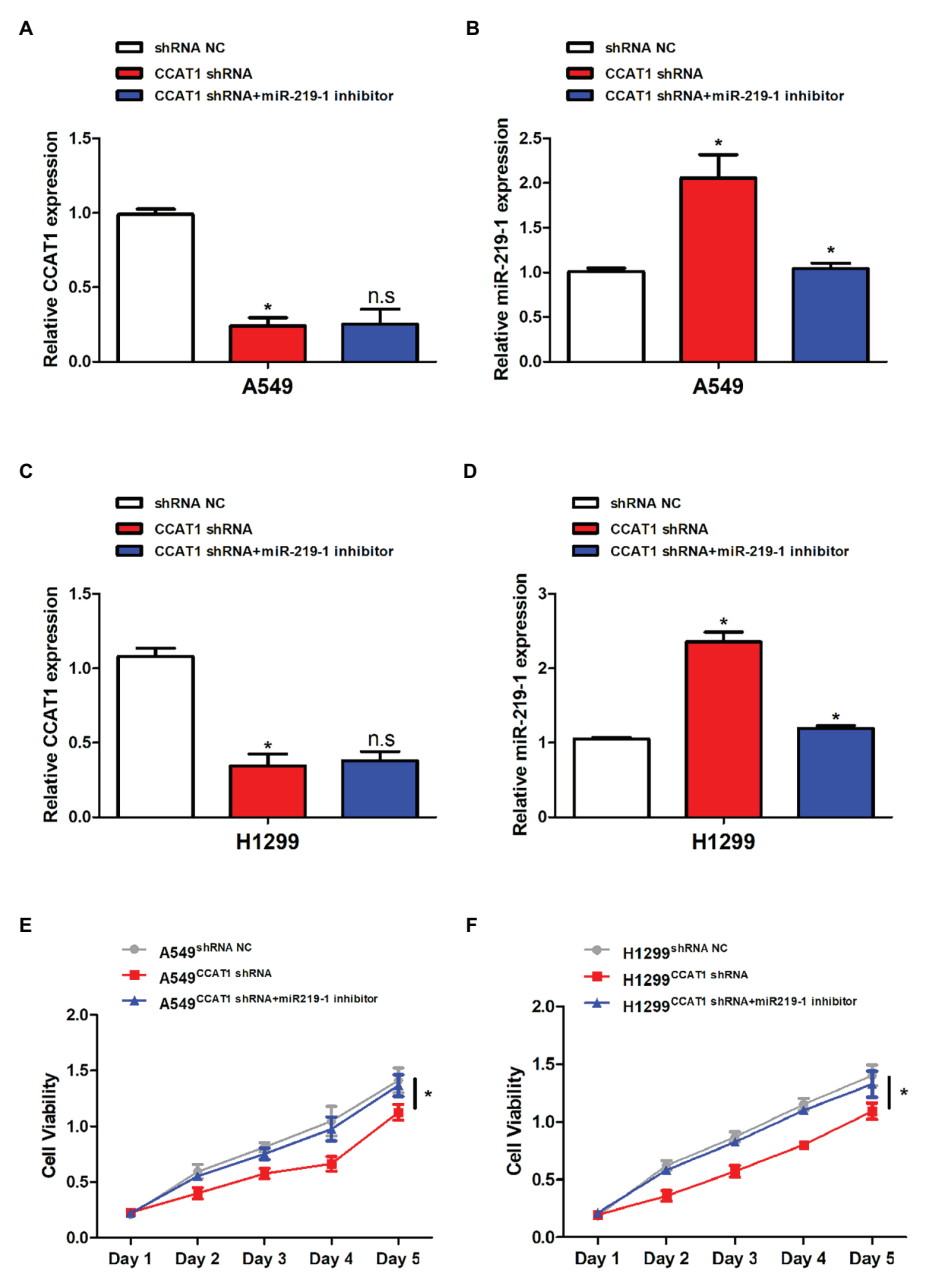
CCAT1 promoted cell proliferation by targeting miR-219-1. **(A,C)** The relative expression of CCAT1 in A549 and H1299 cells transfected with shRNA NC, CCAT1 shRNA, and CCAT1 shRNA+ by the miR-219-1 inhibitor by quantitative real-time PCR (qRT-PCR). **(B,D)** The relative expression of miR-219-1 in A549 and H1299 cells transfected with shRNA NC, CCAT1 shRNA, and CCAT1 shRNA+ by the miR-219-1 inhibitor. **(E,F)** The effects of CCAT1 knockdown on cell proliferation analyzed by CCK-8 assay. Data are shown as means ± SD. NS, not significant, ^*^p < 0.05.

CCAT1 shRNA was used in subsequent experiments. The transfection efficiency was validated by qRT-PCR. Following transfection with CCAT1 shRNA, the CCAT1 expression was significantly decreased while the expression of miR-219-1 was increased in A549 and H1299 cells (Figures 2A–D). Co-transfection with CCAT1 shRNA and the miR-219-1 inhibitor did not affect the expression of CCAT1 and removed the inhibition effect of CCAT1 shRNA on the expression of miR-219-1 (Figures 2A–D). Downregulation of CCAT1 continuously suppressed the proliferation of A549 and H1299 cells, which could be rescued by the miR-219-1 inhibitor (Figures 2E,F). These results indicated that CCAT1 modulated the proliferation of LAD cells by targeting miR-219-1.

### CCAT1 Knockdown Inhibits LAD Cell Migration and Invasion *via* miR-219-1

**Figure 3:**
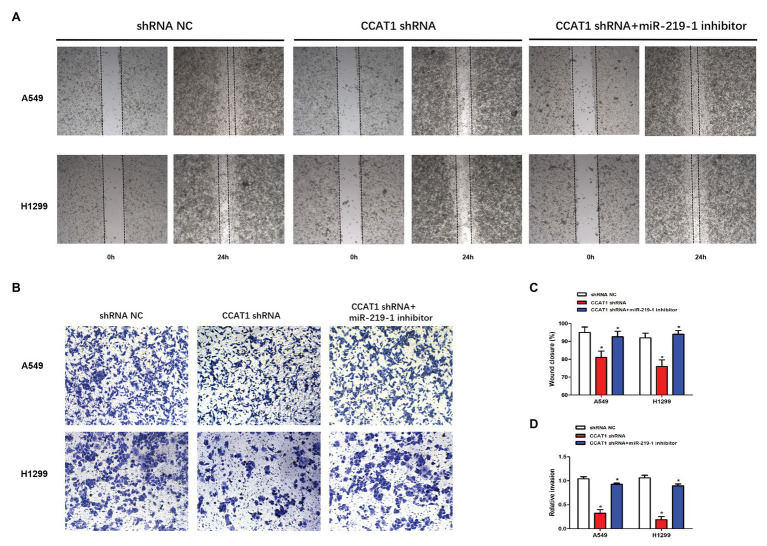
CCAT1 knockdown suppresses the migration and invasion of LAD cells. **(A,C)** The effect of CCAT1 knockdown on cell migration determined by the wound-healing experiment. Pictures were taken at 0 and 24 h. **(B,D)** The effect of CCAT1 knockdown on cell invasion determined by the Transwell invasion assay. ^*^p < 0.05.

To further delineate the roles of CCAT1 and miR-219-1 in the migration and invasion of LAD cells, we used wound-healing and cell invasion experiments. We observed that the migration of A549 and H1299 cells was significantly decreased in the CCAT1 shRNA group compared to the shRNA NC group (Figures 3A,C). Similarly, knockdown of CCAT1 suppressed the invasive capacity of A549 and H1299 cells (Figures 3B,D). Co-transfection with the miR-219-1 inhibitor rescued the effects of CCAT1 on the migration and invasion of A549 and H1299 cells (Figures 3A–D). Taken together, these results suggested that CCAT1 promoted the migration and invasion of LAD cells *via* miR-219-1.

### CCAT1 Knockdown Inhibits Epithelial-Mesenchymal Transition *via* miR-219-1

**Figure 4:**
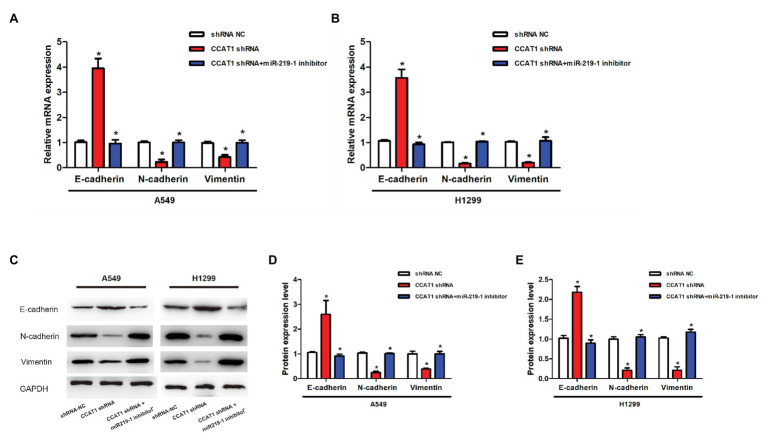
Effects of CCAT1 and miR-219-1 on the expression of protein in LAD cells. **(A,B)** The relative expressions of mRNA of E-cadherin, N-cadherin, and vimentin in A549 and H1299 cells transfected with shRNA NC, CCAT1 shRNA, and CCAT1 shRNA+ by the miR-219-1 inhibitor. **(C–E)** Expression of E-cadherin, N-cadherin, and vimentin determined by western blot. ^*^p < 0.05.

To further explore the effects of CCAT1 and miR-219-1 on EMT, we measured the expression of E-cadherin, N-cadherin, and vimentin by qRT-PCR and western blot. We found that CCAT1 knockdown increased the mRNA expression of E-cadherin but decreased the expression of N-cadherin and vimentin in A549 and H1299 cells, which were reversed by the miR-219-1 inhibitor (Figures 4A,B). Western blot revealed similar changes in the patterns of protein expressions of E-cadherin, N-cadherin, and vimentin (Figures 4C–E). These data suggested that CCAT1 knockdown inhibited the EMT of LAD cells *via* miR-219-1.

### CCAT1 Knockdown Inhibits the Growth of Tumor Xenografts in Nude Mice *via* miR-219-1

**Figure 5:**
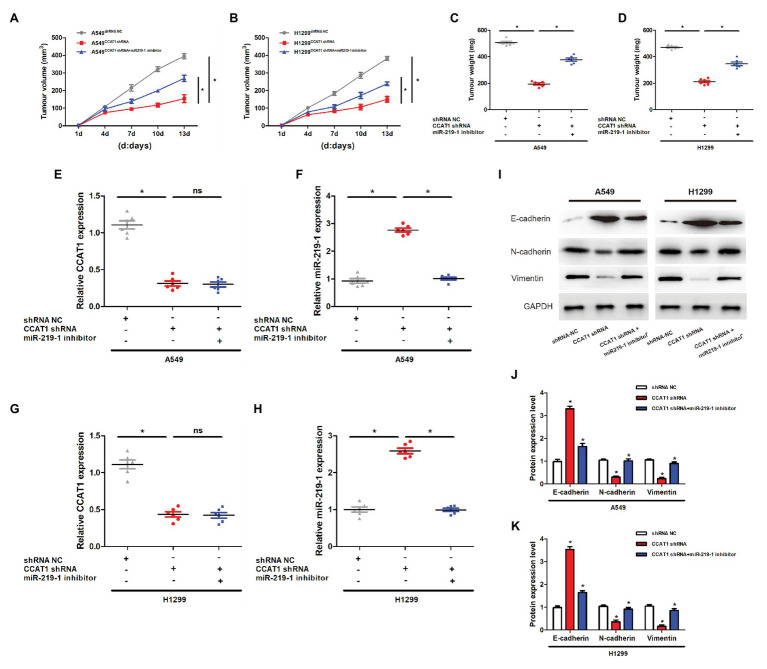
CCAT1 knockdown inhibits the growth of tumor xenografts in nude mice *via* miR-219-1. **(A,B)** Tumor volumes of the xenografts in nude mice. **(C,D)** Tumor weights of the xenografts in nude mice. **(E,G)** The expression of CCAT1 in mice with shRNA NC, CCAT1 shRNA, and CCAT1 shRNA+ by the miR-219-1 inhibitor. **(F,H)** The expression of miR-219-1 in mice with shRNA NC, CCAT1 shRNA, and CCAT1 shRNA+ by the miR-219-1 inhibitor. **(I–K)** Expression of E-cadherin, N-cadherin, and vimentin. ^*^p < 0.05; ns: not significant.

To determine the effect of CCAT1 on tumor growth *in vivo*, we established a nude mice xenograft model by implanting A549 or H1129 cells with the vector CCAT1 shRNA and the CCAT1 shRNA + miR-219-1 inhibitor. We found that knockdown of CCAT1 drastically suppressed the tumor growth of A549 and H1129 cells, which was partially restored by the miR-219-1 inhibitor (Figures 5A,B). Likewise, tumor weights were significantly decreased by CCAT1 knockdown, which was rescued by the inhibition of miR-219-1 (Figures 5C,D). Using qRT-PCR, we observed that the expression of CCAT1 in A549 xenografts was effectively suppressed by the CCAT1 shRNA but was not affected by the miR-219-1 inhibitor (Figure 5E). The expression of miR-219-1 was significantly increased by the CCAT1 shRNA, which was rescued by the miR-219-1 inhibitor (Figure 5F). The same trends were found for H1299 xenografts (Figures 5G,H). Besides, western blot analysis showed that CCAT1 knockdown increased the mRNA expression of E-cadherin but decreased the expression of N-cadherin and vimentin, which were reversed by the miR-219-1 inhibitor (Figures 5I–K). These results indicated that CCAT1 knockdown inhibited the growth and EMT of LAD tumor xenografts by regulating miR-219-1.

## Discussion

It is now well established that lncRNAs are important regulators of human diseases. Previous studies have shown that lncRNA CCAT1 is upregulated in a series of cancers, including colorectal cancer (Nissan et al., 2012), non-small cell lung cancer (Luo et al., 2014), breast cancer (Han et al., 2019), hepatocellular carcinoma (Guo et al., 2019), gastric cancer (Li Y. et al., 2019), gallbladder cancer (Ma et al., 2015), acute myeloid leukemia (Chen et al., 2016), endometrial carcinoma (Yu et al., 2019), nasopharyngeal carcinoma (Dong et al., 2018), and cervical cancer (Shen et al., 2019). In this study, we found that the expression of CCAT1 was significantly increased in LAD tissues than that of adjacent non-tumor tissues. The knockdown of CCAT1 reduced the proliferation, migration, and invasion of LAD cells, which is consistent with a previous study (Luo et al., 2014). Moreover, our *in vivo* experiments showed that knockdown of CCAT1 suppressed the growth of LAD tumor xenografts. These findings indicate that CCAT1 may play a part in the development of LAD.

Accumulating evidence suggests that lncRNAs exert their oncogenic capacities in promoting cell proliferation, migration, and invasion through interacting with miRNAs (Guo and Hua, 2017). The ceRNA hypothesis has proposed that RNA transcripts, such as mRNAs, lnRNAs, and circRNAs, regulate the expression of each other by competitively binding the miRNAs, thus constructing a regulatory network (Salmena et al., 2011). HOXA-AS3 has a significant role in the growth and cell cycle regulation of A549 cells (Zhang et al., 2018). CCAT1 promotes the proliferation and invasion of cervical cancer cells *via* the miR-181a-5p/MMP14 axis (Shen et al., 2019) and promotes endometrial carcinoma *via* miR-181a-5p (Yu et al., 2019). CCAT1 promotes the progression of triple-negative breast cancer by suppressing miR-218/ZFX signaling (Han et al., 2019). CCAT1 boosts gallbladder cancer development *via* sponging miRNA-218-5p (Ma et al., 2015). In acute myeloid leukemia, CCAT1 could upregulate c-Myc through its competing effects on miR-155 (Chen et al., 2016). CCAT1 accelerates the proliferation, migration, and invasion of oral squamous cell carcinoma *via* inhibiting miR-181a (Li G. et al., 2019). In our study, bioinformatics analysis identified miR-219-1 as a binding partner for CCAT1. miR-219-1 was negatively regulated by CCAT1 in LAD cells. Besides, miR-219-1 counteracted the effects of CCAT1 on cell proliferation, migration, invasion, and tumor growth. Together, these results illustrate the importance of miR-219-1 in the CCAT1-mediated growth and metastasis of LAD.

EMT is a process by which a cell transforms from a polarized epithelial phenotype into a mesenchymal phenotype. EMT is featured by the loss of E-cadherin and enhanced expression of N-cadherin, fibronectin, and vimentin. In light of the current evidence, EMT programs are integral components of the malignant progression of all types of carcinoma, including breast, pancreatic, lung, colorectal, hepatocellular, and bladder (Dongre and Weinberg, 2019). The knockdown of lncRNA TTN-AS1 could lead to a significantly higher expression of E-cadherin and lower expressions of N-cadherin, Twist, Snail, and ZEB1 in LAD cell lines (Jia et al., 2019). LncRNA FAM83A-AS1 is found to promote LAD cell proliferation, migration, invasion, and EMT *via* targeting miR-150-5p (Xiao et al., 2019). In non-small cell lung cancer, lncRNA linc00673 could regulate the expression of ZEB1, a key regulator in EMT (Lu et al., 2017). A previous study observed that knockdown of CCAT1 induced a marked reduction in fibronectin and vimentin but upregulated E-cadherin, thereby restoring the epithelial phenotype of H1975 cells, which failed to consider the underlying mechanism of the effects of CCAT1 on EMT (Luo et al., 2014). miR-219 is increasingly recognized as a tumor suppressor in gastric cancer (Li Y. et al., 2019), hepatocellular carcinoma (Gong et al., 2019), and ovarian cancer (Xing et al., 2018). The role of miR-219-1 in LAD was unclear. Our *in vitro* and *in vivo* experiments confirmed that CCAT1 knockdown enhanced the expression of E-cadherin and decreased the expression of N-cadherin and vimentin, which can be rescued by the miR-219-1 inhibitor. Our findings fill the missing link between CCAT1 and EMT by introducing miR-219-1. Moreover, LAD patients with higher CCAT1 expression had a markedly lower overall survival time (Luo et al., 2014). Future epidemiological studies are warranted to assess the value of CCAT1 in predicting the prognosis of LAD patients.

## Conclusion

This study has provided an insight into the roles of CCAT1 and miR-219-1 in LAD progression. CCAT1 promotes the proliferation, migration, invasion, and EMT of LAD cells by sponging miR-219-1. Knockdown of CCAT1 inhibited the growth of tumors *in vivo*. Targeting the CCAT1/miR-219-1 pathway may be a strategy for developing novel LAD treatments. Future work is required to understand the implications of CCAT1 in LAD.

## Data Availability Statement

All datasets presented in this study are included in the article/supplementary material.

## Ethics Statement

The studies involving human participants were reviewed and approved by the Ethics Committee of Henan Provincial Chest Hospital. The patients/participants provided their written informed consent to participate in this study. The animal study was reviewed and approved by the Animal Care and Use Committee of the Huai’an Second People’s Hospital.

## Author Contributions

WW, LyG, and LlG designed the study. WW, ZH, and CW collected the samples and performed the experiment. WW and ZH analyzed the data. WW, LlG, and LlG drafted and revised this manuscript. All authors contributed to the article and approved the submitted version.

### Conflict of Interest

The authors declare that the research was conducted in the absence of any commercial or financial relationships that could be construed as a potential conflict of interest.
